# Assessing Human Eye Exposure to UV Light: A Narrative Review

**DOI:** 10.3389/fpubh.2022.900979

**Published:** 2022-07-06

**Authors:** Michele Marro, Laurent Moccozet, David Vernez

**Affiliations:** ^1^Computer Science Center, University of Geneva, Geneva, Switzerland; ^2^Center for Public Health and Primary Care Medicine (Unisanté), University of Lausanne, Lausanne, Switzerland

**Keywords:** ocular exposure, ultraviolet radiation, UV, eye, ocular dosimetry

## Abstract

Exposure to ultraviolet light is associated with several ocular pathologies. Understanding exposure levels and factors is therefore important from a medical and prevention perspective. A review of the current literature on ocular exposure to ultraviolet light is conducted in this study. It has been shown that ambient irradiance is not a good indicator of effective exposure and current tools for estimating dermal exposure have limitations for the ocular region. To address this, three methods have been developed: the use of anthropomorphic manikins, measurements through wearable sensors and numerical simulations. The specific objective, limitations, and results obtained for the three different methods are discussed.

## 1. Introduction

The obvious increase in environmental ultraviolet radiation (especially the range of UVB, 280–315 nm, and UVA, 315–400 nm) that we have been witnessing for some years has also aroused interest the fields of ophthalmology and optometry (1). Adverse effects could result from excessive exposure in this wavelength range, and several studies have found that a variety of diseases occur from excessively high values of energy absorbed in the ultraviolet range by ocular tissues (1). Of course, artificial UV light is not exempt from this negative effect on human health (2).

The ambient irradiance conditions are determinant in the ocular exposure, but the relationship between the ambient UV intensity and the intensity of light received in the eye is unfortunately not straightforward. The eye is a spherical (aspherical or bi-spherical) and obstructed surface, oriented (most of the time) vertically. Previous studies have indeed shown that ambient irradiance, often represented by the UV index (UVI), is an inadequate predictor of the ultraviolet radiation exposure of eye (3).

The UV index, according to the World Health Organization (WHO), is identified by a number representing the level of UV radiation, therefore the possible risk of developing sunburn or sun erythema of the skin, more or less severe, during a certain exposure time. The UV index is expressed as a function of time and location, so much so that it has become a forecast of risk scenarios for the public. It is determined from a measure (or estimate) of the amount of environmental irradiance by weighting the UV frequency spectrum according to the sensitivity of the human skin (erythemal spectrum) (4). It is clear that this index is not developed to ascertain (or predict) a possible harmful scenario for the eye in an arbitrary external situation. Previous studies evidenced that a situation that is not risky in terms of adverse effects to the skin (such as sunburns) could still be risky for the eyes.

In addition to the biophysical reason, i.e., that the UV index refers to the skin sensitivity and not eye sensitivity, there is also a second reason, of a geometrical nature explaining why ambient irradiance is a poor indicator of eye exposure. In the vast majority of cases, the UV index is calculated from the irradiance spectrum measured on the surface of the earth (5), which is not representative of the anatomical zones of the human body, especially the orientation (mainly vertical) and geometry of the eye.

In this regard, Hatsusaka et al. (3) define an ocular UV index (OUVI), i.e., a specific UV index for eyes. Through measurements, obtained with an anthropomorphic manikin, Hatsusaka et al. relate the environmental irradiance with the ocular irradiance and defines, by means of a simple linear regression, a formula that allows to calculate OUVI (using the same scale of the UVI) directly from the ocular UV irradiance. Comparing the UVI and the OUVI, the same index was noted around midday in summer, whereas in the morning and the afternoon a higher level of OUVI is registered (an average value of 3.7 vs. 2.5). During the winter, when UVI values are generally low (maximum index of 1), the OUVI instead records values up to 4.

A number of parameters must be considered to understand eye exposure: how much light it receives, how it is distributed over the ocular surface and how the eye changes as a function of environmental conditions. These are generally assessed by direct or indirect measurements of the ocular dose received. Measuring the ultraviolet radiation (UVR) eye dose is a significant challenge as it depends on several factors that are not always easily measurable or repeatable in experiments, and above all cannot be parameterized using empirical formulas. It is necessary to consider that this value has, above all, a great anatomical-geometric dependence [as has already been investigated in numerous works, such as that of Sliney (6, 7)]: the surface of the eye is indeed an obstructed surface which has a sensitive part whose surface changes continuously.

Physiological phenomena such as squinting and different blink frequencies impose, moment by moment, a variation of the sensitive surface exposed to light. Recently, the filtering role of the eyelashes and how they can reduce the intensity of ultraviolet light received by the eye depending on the direction of the incoming light has been investigated (8). Furthermore, the rotation of the eyeball have also obvious repercussions on the amount of radiation received inside the eye (9). The rotation of the head imposes a further degree of complexity to the phenomenon, as there is no particular pattern followed but always a series of random directions that change moment to moment. Environmental factors [as pointed out in (10, 11)], such as the amount of reflected light, which in turn depends on the local albedo conditions, influence the determination of the effective exposure. Although there are no specific studies on this subject to the author's knowledge, it is generally hypothesized that eyebrows reduce the intensity of light reaching the eye, especially for sufficiently large angles of the sun's elevation. The possible role of hair cannot be ruled out either. Experimental studies have shown that hair acts as a protective agent against UV radiation (12, 13).

The aim of this paper is to provide a narrative review based on the research question: *how does ultraviolet light reach our eyes?* This question is addressed through a narrative review, aiming at understand and study all the variables affecting this exposure. This research focuses on works found in the literature that aim to measure the ocular exposure to ultraviolet light and study the influence of the environmental and anatomic exposure determinant. Both ambient and artificial sources of UV light were included, although the latter are limited. The study of the relationship between the level of environmental UV and ocular exposure, also excluding studies that specifically investigate the effect of UV radiation absorption on ocular tissues, is generally complex and difficult to determine. The variables that influence this measure are in general greater in number than in a laboratory-controlled case. The research question is therefore oriented to understand the factors that determine ocular exposure and the different methods adopted to study these factors.

## 2. Methodology

There are many variables involved in these measurements, as described in the previous section. However, the method used allows the study to focus on specific variables. It imposes certain restrictions on the variable to be investigated, therefore a grouping that was quite efficient was by method.

The research strategy followed in producing this article is that of a narrative review. Eligibility criteria were defined such that articles related to UV light that specifically addressed the human eye or ocular region were included. Consequently, articles that did not treat UV light and that did not focus on the human eye were excluded.

The articles were first selected by title. Abstracts and conclusions were read for the selected articles, leading to a second selection. Finally, the complete reading of the articles led to the creation of a final set. The literature cited by the selected articles was analyzed with the aim of finding other articles related to the research question. In addition, some articles were selected since they were considered relevant as examples and to show further methods and application. The entire research, analysis, reading and selection of articles was carried out by a single person. The databases used for this research were: *Web of Science, Google Scholar, PubMed*, and *IEEE Xplore*. The keywords used were *(radiation) ocular exposure, ocular irradiance, ocular ultraviolet radiation, eye (ultraviolet) exposure*.

**Figure 1:**
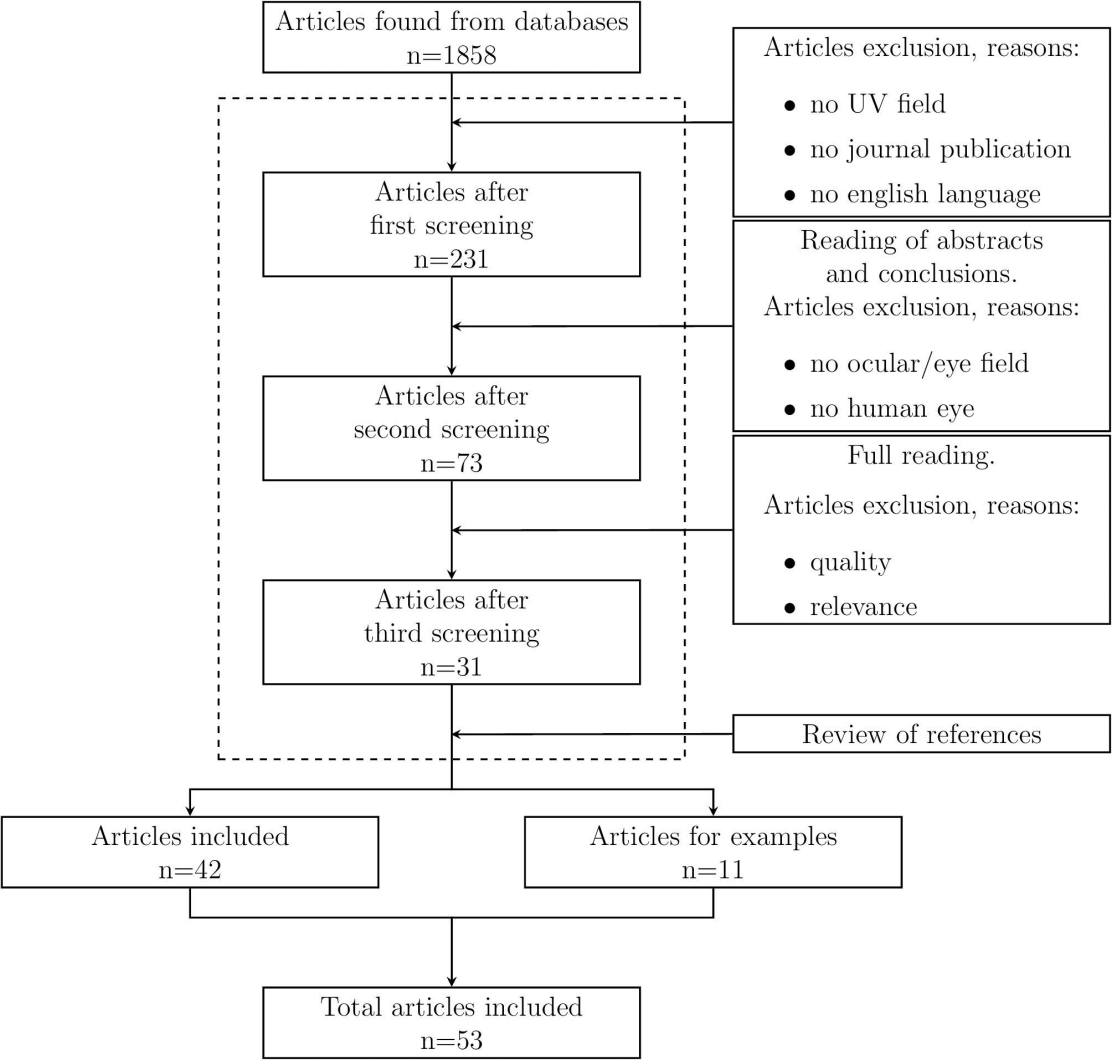
Flow diagram of the review process performed in this review.

The process followed in this research strategy is schematized in Figure 1 as flow diagram.

## 3. Measuring Ocular Exposure With Anthropomorphic Manikins

A fairly widely used method to determine the amount of light reaching the ocular area, as well as the distribution of light as a function of daily parameters such as the angle of elevation of the sun, is through the use of anthropomorphic manikins [such as in (14–18)]. The experimental setup is almost always the same and consists of using a light sensor inserted into the eye area of the manikin and then exposing the manikin to sunlight for an arbitrary period of time. This measurement does not represent the amount of light arriving on the cornea (which would undoubtedly be more interesting), but the light arriving on the ocular area. This is because a light sensor is generally a flat surface, which does not represents the aspherical (or bi-spherical) surface of the human cornea. Typically, the surface of the sensor coincides with the apex of the cornea, so that the surface is tangent to it.

The anatomy of the manikin plays an important role in this method, since different manikins' morphologies will cast different shadows for different solar elevation angles. This was evidenced by the work of Chen et al. (19) in which two mannequins with typical average facial features of Asians and Europeans demonstrate a different level of ocular exposure caused by the difference in superciliary arch and glabella. However, this is also true for a human population: inter-individual variations in anatomy will undoubtedly influence the results. The position of the light sensor could also influences the final result (although it is sometimes not reported as a decisive factor). Indeed, considering possible variations for the optical axis alone, in a hypothetical situation where the sensor is partially covered by a shadow cast by a part of the face (e.g., the nose) at a relatively large angle to the normal of the sensor surface, this variation could lead to considerable differences in the results. The same concept also applies to variations in position in the plane normal to this axis. These two sources of error make the results found in the different works difficult to compare (whether a comparison should be necessary or not). However, given the relative ease of the experiment, as well as the similarity of the facial anatomy profile, it is possible to find a common trend in the results. It is interesting to note, however, that none of the studies identified with manikins mentions the issue of light reflection as a geometric factor that could affect the final result, for example by altering the reflectance value (20).

The method of manikins used to measure ocular radiation has some rather obvious limitations. First, it does not record a typical daily ocular exposure to light, which is certainly much more complex to determine, but the result in relation to the angle of the head and the height of the sun above the horizon. Second, it does not record the complex influence of phenomena such as squinting or change of head direction on the effective exposure. In about half of the studies reviewed, the manikin's head is orientated with a downward frontal angle (usually 10° or 15° below the horizon) to represent a realistic situation of a person who is walking. Some studies rotate the manikin by a complete tour on itself around the horizontal plane to determine the same result from all head orientation, as in (21–26). Considering, that the time of rotation on the manikin on itself is less than the time necessary for a significant variation in the displacement of the sun in the sky (and therefore also of its irradiance), this rotation allows a richer data recording for a given elevation angle of the sun. Instead, some studies point the manikin always toward the sun, as in (27).

Some studies conducted with manikin method are intended to investigate the amount of light reaching the eye and its dependence to environmental parameters as well as facial anatomy, without consideration to health or prevention issues. Predicting the effect of the solar elevation angle the resulting seasonal variation is, for instance, not straightforward. Studies with anthropomorphic manikins showed a similar pattern among different experiments, namely that of a bimodal distribution. It is usually shown during warm seasons, when the solar elevation angle becomes high enough to prevent direct radiation from the sun from hitting the eye. In this particular time interval, it can be seen that during a daily exposure, while the ambient radiation increases with the solar elevation angle, there is no increase in the received ocular radiation, but rather a decrease. Indeed, the radiation that reaches the eye (the light sensor) in this time interval is only due to the sum of the diffuse radiation and the reflected radiation from the ground surface (albedo). This profile occurs because of the superposition of two effects: the anatomy of the ocular area and the orientation of the receiving surface of the eye. The bimodal distribution was also observed for measurements that did not directly target the eye, but other parts of the head, in particular the cheeks and the nose, thus vertically oriented surfaces. For example, in Wang et al. (25, 28) the ambient irradiance is measured on various parts of the face using a manikin. Bimodal distribution was observed for sensors located on the cheeks, nose tip and forehead, indicating that the orientation of these surfaces plays a key role in determining the risk factor. The profile of the bimodal distribution changes throughout the year. Generally, it is not observed in the cold season, as the sun does not reach sufficiently high elevation angles.

In other words, at equal intensity we receive more direct light during the months (or in locations) where the sun does not reach high elevation angles. The discussion becomes more complicated if we consider that the intensity of ambient UV light changes throughout the year and generally reaches its maximum value in summer. Sasaki et al. (29) compared the total exposure received by the eye area of a manikin exposed on 21 September and then on 21 November, both times facing the sun. The daily UV intensity in September was only 8% higher than in winter despite an almost 30% decrease in daily ambient UV intensity. Daily eye exposure during the solstices and equinoxes were also recorded by (23). Bimodal distribution was observed for autumn, spring, and summer. Notably, for the latter season, ultraviolet exposure values were higher in the morning and evening, rather than around midday, when the ambient UV intensity is at its highest. Furthermore, the highest daily ocular exposure value recorded appears to be in winter.

The manikin method was also used to determine the level of UVR received by the ocular region in an indoor situation by varying the orientation and distance of the manikin in a room relative to a window (30).

Anthropomorphic manikins have also been used to study the level of protection offered by objects developed for this very purpose, such as sunglasses and hats, as in (31). This is also the case in (32), in which two manikins (heads) on which UV sensors have been placed are used to determine the level of protection offered by a hat. Meanwhile, in (33), a manikin is used to measure UV exposure in sitting and standing positions.

## 4. Measuring Ocular Exposure With Wearable Sensors

Another method used to measure ocular exposure is to fit light sensor and then exposing the carrier in a given scenario. In this regard, Fleming et al. (34) developed a particular device consisting of five UV sensors. This apparatus was mounted on a spherical plastic shell of a size that could be superimposed on the eye (with the eyelids lowered). Wearable sensors are always used for preventive purposes, aiming at establishing the amount of UV rays received by the front ocular surface for different solar elevation angles. In this case, these five sensors together, which cover the eye from the nasal to the temporal-central area of the eye, have a greater field of view than a flat-sensing-part single sensor. This feature makes this method versatile, as it can measure the UV radiation in different areas of the ocular region and record, for example, the UV absorbed by the nasal limbus. Similarly to the manikin method, it involves measuring ultraviolet radiation using different head orientations. The sensor is, however, not used in situations where the subject can move, which brings to this method the limitations already seen for the manikin method. The same method was previously used by (20) to investigate the influence of ultraviolet radiation reflected by the skin of the nose. In this case, the experiment was carried out under controlled lighting conditions by using a diffuse artificial light source to illuminate the five sensors. Walsh observed an increase in UVR on the nasal side of the eye due to light reflection of the nose.

A similar method, but with an even more ambitious goal, is to measure ultraviolet radiation directly on the surface of the eye. This solution, presented by Sydenham et al. (35), uses polysulphone contact lenses, which degrade when exposed to ultraviolet radiation. This material had already been widely used for dosimetric measurements, but never in the form of contact lenses. Knowing the dose-response between UV light and polysulphone lenses degradation, it is possible to quantify exposure by comparing the absorption of the lens before and after exposure by spectrophotometry. Because of the material of which these lenses are made, the measurement time was limited to a maximum of one hour, although it is feasible to extend this time with specific adapters. The results, expressed in terms of Ocular Ambient Exposure Ratio (OAER), are compatible with measurements found in previous works. The same method was used McLaren et al. (36) (of which Sydenham is in fact a co-author). In this study the contact-lens method was applied to measure the ocular UV dose received by two subjects during a winter day. Differences were found between McLaren et al.'s and Sydehham et al.'s results, but these were attributed to differences in the experimental setup.

Unfortunately, no recent studies have been found on dosimetric measurement using contact lenses specifically for UV radiation. Instead, this method is currently used, with some refinement and improvement, in the field of diagnostic radiology, thus using ionizing radiation. In this respect, we cite, as an example, the work of Park et al. (37) in which contact lenses made of acrylic material were developed for *in vivo* measurements of the dose received during radiotherapy sessions. Similarly, Kim et al. (38) proposed contact lenses for *in vivo* dosimeter measurements in the field of radiation therapy. Compared to the anthropomorphic manikin method, the main advantage of the contact-lens method is more realistic dose measurement, theoretically closer to the true exposure value. This is because contact lenses do not need a flat measurement surface, as do light sensor, nor do they have the same sources of error with regard to positioning (although for contact lenses it becomes important to quantify lens rotation during the measurement period). The contact-lens method could also be assumed to reflect better the effective ocular dose received by an individual since it take into account dynamic effects, such as eyes and eyelids movements. When using manikins, one determines the dose received by the eyes in a given static situation, which do not vary during the measurement. While the manikins can often rotate horizontally, which makes for interesting measurements, the final result is still far from a true exposure received by a human eye. Contact lenses allow researchers to quantify the ocular exposure to light in a typical outdoor situation, enriching the final data with all those processes that are difficult to quantify or emulate in manikin, such as squinting, change of head direction, and blinking.

Some studies propose alternative methods to both the manikin or contact lenses, for example Duncan et al. (39–41). These studies combine the measurement of the eye dose by means of a light sensor and a mathematical model for the determination of the level of exposure received during a certain time period. Similarly to Sydenham et al., it is based on the concept of the OAER, defined as follows


(1) $$R_{oa}=E\left\{\frac{\int^{\text{​}}f_{T}(t)E_{a}(t)dt}{\int^{\text{​}}(f_{T}(t)/f(t))E_{a}(t)dt}\right\}.\;\;$$

Where *E* is the expectation operator, *E*_*a*_(*t*) is the global environmental exposure ratio, *f*(*t*) is the fraction of global environmental exposure that hits the plane tangent to the apex of the cornea and *f*_*T*_(*t*) is the time spent outside, which is 0 in the case where no exposure occurs. The OAER is calculated from measurements taken using light sensors (some developed for this purpose) worn by a population of individuals, which record UV (and visible) radiation in the tangent plane to the face and ambient UV (and visible) radiation. This quantity makes possible to determine the personal exposure from the estimated exposure according to the formula:


(2) $$H_{p}=NR_{oa}\left[\sum_{i}F_{t}(t_{i})Q_{a}(t_{i})\right]T_{hat}T_{eye}G.\;\;$$

Where *N* is the number of days, *F*_*t*_(*t*_*i*_) is the average fraction of time spent outside for the time interval *t*, *Q*_*a*_(*t*_*i*_) is the average environmental exposure during the same time interval, *T*_*hat*_ and *T*_*eye*_ are correction factors that take into account the presence or absence of hats and glasses, respectively, and *G* is the geographical correction factor, which takes into account ozone and cloud cover (measured by satellites). The type of measurement performed and the sensor used may constitute limitations of this method, as it does not measure the UV radiation arriving on the eye surface or take into account blinking, squinting and light reduction due to eyelashes and eyebrows. Other types of limitations, however, were resolved using interviews, such as for the quantification of time spent wearing hats and glasses (terms *T*_*hat*_ and *T*_*eye*_ in the Equation 2). This method undoubtedly has advantages over other methods that used solely interviews, without any kind of measurement, as in (42), where UVR exposure is estimated from man-made (welding) sources, using a simple three-index system (numbers of workers exposed, time of exposure, and intensity of exposure).

In this regard, a number of works on the investigation of ocular exposition of welders were carried out, even though the measurements do not focus directly to the eye (43). The methods already reported in this section (e.g., using often polysulphone films) were used in this filed for determining the dose received from this artificial source.

A concept similar to Duncan's method involving the use of questionnaires and a mathematical model based on the OAER, but without corrective parameters, was also applied to a population study in (44). A slightly different formulation was described and used instead in (45) in which now Equation (2) is used for a longer period of time by taking into account the monthly variability of the geometric correction term and the OAER, and the daily variability of all other terms. Such method was used to determine the ambient UV ocular exposure for a population in order to study the relation between UVB and lens opacities.

Approaches which are similar but do not implement a mathematical model, have been used to assess exposure to other parts of the body. In (46), a sensor was used to determine exposure for several subjects during their usual activities (children, lifeguards, and mountain guides). In (47), a sensor placed vertically and attached laterally to the head was used to record the UV exposure of mountain guides over a period of one year.

## 5. Estimating Ocular Exposure With Numerical Simulations

A distinction must be made among the different types of numerical models used to determine the radiation dose received by the eye. There are two main types, and each has different purposes and applications.

- Tissues dosimetry models
  The most widely used models simulate ionizing radiation, i.e., short-wave radiation (mainly X-rays and gamma rays) that according to ISO standards conventionally also cover part of the ultraviolet range, specifically the part of this range where the wavelengths are shorter. These models, which are also probably the most used, are mainly used for medical applications, to determine by numerical simulation the dose to the eye (or some specific part of it) during a radiotherapy (or similar) session. Such models typically use Monte Carlo simulation techniques to simulate the path of many photons and their interaction with matter. For example, in Carinou et al. (48) numerical Monte Carlo simulations are performed to emulate radiology and cardiology sessions using virtual manikins. In Caracappa et al. (49), a detailed multi-resolution eye model was developed and then inserted into a virtual manikin to simulate the dose received from a source between 10^−2^ and 10 MeV. In a more recent paper, Santos et al. (50) implemented a multi-resolution model to determine the best solution for an eye dosimetry model. The number of studies dealing with ocular dosimetry simulation of ionizing radiation is substantial. We will not detail them further here, as it is beyond the scope of this review.
- Surface models
  In contrast to the tissues dosimetry models, these models simulate light exposure at the body surface, involving macroscopic variables, such as irradiance and radiant dose. They do not consider either individual photons or their interaction with matter, but only the dose absorbed by a single surface, more or less complex.

In the case of the tissue dosimetry models, the simulation emulates an indoor laboratory situation while typically in surface models an outdoor situation is analyzed and subjected to illumination by ambient UV radiation. These outdoor setting also differs in its spatial distribution. Furthermore, for tissues dosimetry models the simulation is usually static, i.e., the parts subjected to the treatment with ionizing rays are fixed during the simulation time. For surface models this aspect is not always present, since the aim is to best represent a dynamic situation, where the body position will change during the simulation period. For this reason, surface models concentrate on the simulation of cumulative variables, such as the radiant dose, expressed as a function of periods of many hours (or days or even more) of exposure, unlike the tissues dosimetry models. The behavior of the surface models approximately emulates approximately the situations described above with manikins and light sensors. Surface models have been developed and used to numerically estimate the exposure to natural ultraviolet light for different situations. In general, there are no models developed specifically for ocular exposure, but models to determine skin exposure. In many cases, numerical models that simulate light exposure of the skin are in some way usable or adaptable to simulation for ocular exposure, so they will be reported here when deemed adaptable to the ocular exposure. Perhaps, the greatest challenge of these numerical models has been to find a way to parameterize the complex outer surface of a person, since they are intended to simulate the radiation received by different parts (anatomical zones) of the human body. A typical research question answered by these simulations is *how does the total radiation received for a given period of time is distributed over the whole body?*

A versatile solution to this problem of parameterizing the surface of the human body was presented by Streicher et al. (51). In this study, the complex geometry of the problem is represented by a set of flat triangles, arranged in space and oriented to approximate the real surface. Together with this three-dimensional object, it is possible to simulate the path of the sun during an arbitrary period of time and, by means of ray-tracing techniques, to calculate the portion of energy received by each triangle. The model also takes into account the diffuse component of the sunlight (which is mandatory for simulations of this type), and calculates the sky view factor for each triangle, without taking into account ground reflection, thus assigning an albedo value of 0. The results are presented for 20 different anatomical zones of the human body, obtained by averaging over defined sets of triangles that compose the various zones.

The same methodology was used by Vernez et al. (52), who present a numerical model (SimUVEx, from Simulation of solar UltraViolet Exposure) able to use real solar irradiance data to simulate the exposure received on the skin. Here too, the surface of the human body is represented by a set of triangles, described as coordinates in space stored in a single text file. This model also takes into account the radiation reflected locally from the earth's surface, modeling it as a Lambertian source. The model, which was later improved to also implement diffuse anisotropic radiation, has been used to investigate UV radiation received in various scenarios. For example, SimUVEx was used in the work of Backes et al. (53) to calculate exposure in the ocular region with various types of glasses. Subsequently, an even more updated version of SimUVEx, which features several improvements, was used in (8), specifically for the eye.

## 6. Conclusions and Future Perspectives

This review summarizes the current publications concerning ocular exposure to ultraviolet light. Quantifying the intensity of this radiation received by the eyes, and further how this is distributed, is a challenge that can be addressed with different methods. The methods reported in literature, along with their limitations, are highlighted in this study. Along with that their limitations, their potential and possibilities are also described, marking the particular goal and application of each method. They were grouped into three categories: the method of anthropomorphic manikins, the method of wearable sensors, and the method of numerical simulations. Each assessment method identified shed light on certain aspects of ocular exposure and their implications for health, exploring several research questions.

The manikin method makes it possible to study how and how much light is received in the ocular region in different situations of illumination and protection. However, such method cannot be used to determine the ocular exposure to ultraviolet light for a realistic exposure situation: eyeball rotation, head rotation, squinting, and blinking are some examples of phenomena that cannot be included in the measurement.

Some methods were shown to be more appropriate to mimic realistic exposures. This is the case of the contact-lens method, although the last application found is more than twenty years old. This rarity may be related to the increasing complexity of human subject studies, which tends to make simulation studies more popular. Other types of measurement focused instead on longer periods of time, at the expense of accuracy, estimating the OAER for general populations. This method could potentially be used anywhere, knowing the various factors that influence the particular geographical area and daily habits (i.e., time spent outside).

Finally, it was noted that the numerical simulations method is not widely used in this field, although it proves to be quite promising. Through numerical simulation it is in fact possible to simulate an arbitrary source of light, ambient and artificial, and study in detail how it distributes on an object of any shape. The real challenge is likely simulating a realistic situation, trying to include all the parameters that can influence the result. Regardless, the ability to construct arbitrary scenarios makes this method a potential resource for future research.

## Data Availability Statement

The original contributions presented in the study are included in the article/supplementary material, further inquiries can be directed to the corresponding author/s.

## Author Contributions

MM conducted the entire research, designed the method, performed its application, read and reviewed the articles, and wrote the original draft of the manuscript. LM performed the revision. DV contributed to the final revision and wrote the final draft of the manuscript. All authors contributed to the article and approved the submitted version.

## Funding

This research was developed within the framework of the InExES project: Coupling Internal and External Eye Simulation for a Better Prediction of Natural and Artificial Light Exposure (Internal and External Eye Simulation) which is financially supported by the Velux Stiftung in Zürich, Switzerland (Grant number 1254). Open access funding was provided by the University of Geneva.

## Conflict of Interest

The authors declare that the research was conducted in the absence of any commercial or financial relationships that could be construed as a potential conflict of interest.

## Publisher's Note

All claims expressed in this article are solely those of the authors and do not necessarily represent those of their affiliated organizations, or those of the publisher, the editors and the reviewers. Any product that may be evaluated in this article, or claim that may be made by its manufacturer, is not guaranteed or endorsed by the publisher.

## References

[1] Yam J Kwok A. Ultraviolet light and ocular diseases. Int Ophthalmol. (2014) 34:383–400. 10.1007/s10792-013-9791-x 23722672

[2] Polani Chandrasekar R Ponnaiah M Anandhi D John D. The association between exposure to artificial sources of ultraviolet radiation and ocular diseases in humans: a systematic review protocol. JBI Evid Synth. (2020) 18:1766–73. 10.11124/JBISRIR-D-19-00206 32898369

[3] Hatsusaka N Seki Y Mita N Ukai Y Miyashita H Kubo E . uv index does not predict ocular ultraviolet exposure. Transl Vis Sci Technol. (2021) 10:71. 10.1167/tvst.10.7.1 34061949 PMC8185396

[4] Fioletov V Kerr J Fergusson A. The UV index: definition, distribution and factors affecting it. Can J Publ Health Rev Can Santé Publ. (2010) 101:5–9. 10.1007/BF03405303 21033538 PMC6974160

[5] Madronich S. Analytic formula for the clear-sky UV index. Photochem Photobiol. (2007) 83:1537–8. 10.1111/j.1751-1097.2007.00200.x 18028230

[6] Sliney DH. UV radiation ocular exposure dosimetry. J Photochem Photobiol B Biol. (1995) 31:69–77. 10.1016/1011-1344(95)07171-5 8568605

[7] Sliney D. Geometrical assessment of ocular exposure to environmental UV radiation -implications for ophthalmic epidemiology. J Epidemiol Jpn Epidemiol Assoc. (2000) 9:S22–32. 10.2188/jea.9.6sup_22 10709347

[8] Marro M Moccozet L Vernez D. Modeling the protective role of human eyelashes against ultraviolet light exposure. Comput Biol Med. (2021) 141:105135. 10.1016/j.compbiomed.2021.105135 34959113

[9] Lund SBEEP David J. Retinal injury thresholds for blue wavelength lasers. Health Phys. (2006) 90:477–84. 10.1097/01.HP.0000190115.83416.cb 16607179

[10] Turnbull D Parisi A. Increasing the ultraviolet protection provided by shade structures. J Photochem Photobiol B Biol. (2005) 78:61–7. 10.1016/j.jphotobiol.2004.09.002 15629250

[11] Turner J Parisi AV. Influence of reflected UV irradiance on occupational exposure from combinations of reflective wall surfaces. Photochem Photobiol Sci. (2013) 12:1589–95. 10.1039/c3pp50059d 23677134

[12] Parisi A Smith D Schouten P Turnbull D. Solar ultraviolet protection provided by human head hair. Photochem Photobiol. (2008) 85:250–4. 10.1111/j.1751-1097.2008.00428.x 18764896

[13] de Gálvez MV Aguilera J Bernabó JL Sánchez-Roldán C Herrera-Ceballos E. Human hair as a natural sun protection agent: a quantitative study. Photochem Photobiol. (2015) 91:966–70. 10.1111/php.12433 25682789

[14] Downs N Kimlin M Parisi A McGrath J. Modelling human facial UV exposure. Radiat Prot Aust. (2001) 17:130–5. 10.1034/j.1600-0781.2001.170305.x 25154366

[15] Ono M. Studies on ultraviolet radiation and health effects: ocular exposure to ultraviolet radiation. Dev Ophthalmol. (2002) 35:32–9. 10.1159/000060808 12061277

[16] Kwok LS Kuznetsov VA Ho A Coroneo MT. Prevention of the adverse photic effects of peripheral light-focusing using UV-blocking contact lenses. Invest Ophthalmol Vis Sci. (2003) 44:1501–7. 10.1167/iovs.02-0380 12657585

[17] Hu L Wang F Ou-Yang NN Gao N Gao Q Ge T . Quantification of ocular biologically effective UV exposure for different rotation angle ranges based on data from a manikin. Photochem Photobiol. (2014) 90:925–34. 10.1111/php.12267 24588689

[18] Yu J Hua H Liu Y Liu Y. Distributions of direct, reflected, and diffuse irradiance for ocular UV exposure at different solar elevation angles. PLoS ONE. (2016) 11:e166729. 10.1371/journal.pone.0166729 27846278 PMC5112793

[19] Chen R Wang N Hua H Huang L Li M Zou Z . Optical modeling and physical experiments on ocular UV manikins exposure. IEEE Access. (2019) 7:478–86. 10.1109/ACCESS.2018.2885533

[20] Walsh JE Bergmanson JPG Wallace D Saldana G Dempsey H McEvoy H . Quantification of the ultraviolet radiation (UVR) field in the human eye in vivo using novel instrumentation and the potential benefits of UVR blocking hydrogel contact lens. Br J Ophthalmol. (2001) 85:1080–5. 10.1136/bjo.85.9.1080 11520761 PMC1724131

[21] Wang F Gao Q Hu L Gao N Ge T Yu J . Risk of Eye Damage from the Wavelength-Dependent Biologically Effective UVB Spectrum Irradiances. PLoS ONE. (2012) 7:e5225. 10.1371/journal.pone.0052259 23284960 PMC3527526

[22] Gao N Hu LW Gao Q Ge TT Wang F Chu C . Diurnal variation of ocular exposure to solar ultraviolet radiation based on data from a manikin head. Photochem Photobiol. (2012) 88:736–43. 10.1111/j.1751-1097.2012.01094.x 22268421

[23] Hu L Gao Q Xu W Wang Y Gong H Dong GQ . Diurnal variations in solar ultraviolet radiation at typical anatomical sites. Biomed Environ Sci. (2010) 3:234–43. 10.1016/S0895-3988(10)60058-X 20708504

[24] Hu L Gao Q Gao N Liu G Wang Y Gong H . Solar UV exposure at eye is different from environmental UV: diurnal monitoring at different rotation angles using a manikin. J Occup Environ Hyg. (2013) 10:17–25. 10.1080/15459624.2012.737700 23145494

[25] Wang F Ge T Gao Q Hu L Yu J Liu Y. The distribution of biologically effective UV spectral irradiances received on a manikin face that cause erythema and skin cancer. J Photochem Photobiol B Biol. (2014) 140:205–14. 10.1016/j.jphotobiol.2014.08.004 25169771

[26] Wang F Liwen H Gao Q Gao Y Liu G Zheng Y . Risk of ocular exposure to biologically effective UV radiation in different geographical directions. Photochem Photobiol. (2014) 90:12287. 10.1111/php.12287 24804634

[27] Hua H Hu L Chen R Wang N Ma F Li X . Solar ultraviolet exposure and absorbed irradiance of the cornea and anterior chamber/lens: a monitoring model using porcine eyes in a manikin. IEEE Access. (2020) 8:623–32. 10.1109/ACCESS.2019.2961704

[28] Wang F Ming YJ Qi YD Qian G Hui H Yang L. Distribution of facial exposure to non-melanoma biologically effective UV irradiance changes by rotation angles. Biomed Environ Sci. (2017) 30:113. 10.3967/bes2017.015 28292349

[29] Sasaki H Sakamoto Y Schnider C Fujita N Hatsusaka N Sliney D . UV-B exposure to the eye depending on solar altitude. Eye Contact Lens. (2011) 37:191–5. 10.1097/ICL.0b013e31821fbf29 21670696

[30] Hua H Chen R Yang T Yang D Deng Y Liu Y. Quantification of indoor ocular exposure to solar ultraviolet light with four room orientations: using a model monitor embedded in a manikin head. IEEE Access. (2020) 8:13387–404. 10.1109/ACCESS.2020.2966687

[31] Deng Y Zhang C Zheng Y Li R Hua H Lu Y . Effect of protective measures on eye exposure to solar ultraviolet radiation. Photochem Photobiol. (2021) 97:205–12. 10.1111/php.13327 32875566

[32] Downs N Parisi A. Patterns in the received facial ultraviolet exposure of school children measured at a sub-tropical latitude. (2008) 84:90–100. 10.1111/j.1751-1097.2007.00203.x 18173708

[33] Parisi A Kimlin M Lester R Turnbull D. Lower body anatomical distribution of solar ultraviolet radiation on the human form in standing and sitting postures. J Photochem Photobiol B Biol. (2003) 69:1–6. 10.1016/S1011-1344(02)00385-8 12547490

[34] Fleming D Walsh J Moore L Bergmanson J McMahon D. A novel sensor array for field based ocular ultraviolet radiation measurements. Radiat Protect Dosimet. (2006) 118:265–74. 10.1093/rpd/nci346 16192325

[35] Sydenham MM Collins MJ Hirst LW. Measurement of ultraviolet radiation at the surface of the eye. Investigative *Ophthalmol Vis Sci*. (1997) 38:1485–92. 9224276

[36] McLaren K Watson W Sanfilippo P Collins M Sydenham M Hirst L. Contact lens dosimetry of solar ultraviolet radiation. Clin Exp Optomet. (1997) 80:204–10. 10.1111/j.1444-0938.1997.tb04884.x

[37] Park JM Lee J Kim H Ye SJ Kim Ji. Development of an applicator for eye lens dosimetry during radiotherapy. Br J Radiol. (2014) 87:20140311. 10.1259/bjr.20140311 25111733 PMC4170865

[38] Kim Ji Cho J Son J Choi CH Wu HG Park JM. Contact lens-type ocular *in vivo* dosimeter for radiotherapy. Med Phys. (2019) 47:722–35. 10.1002/mp.13932 31743441

[39] Duncan DD Schneider W West KJ Kirkpatrick SJ West SK. The development of personal dosimeters for use in the visible and ultraviolet wavelength regions. Photochem Photobiol. (1995) 62:94–100. 10.1111/j.1751-1097.1995.tb05244.x 7638275

[40] Duncan DD Muñoz B Bandeen-Roche K West SK. Visible and ultraviolet-B ocular-ambient exposure ratios for a general population. Salisbury Eye Evaluation Project Team. Invest Ophthalmol Vis Sci. (1997) 38:1003–11. 9112996

[41] Duncan DD B M SK W Team SEEP. Assessment of ocular exposure to visible light for population studies. Dev Ophthalmol. (2002) 35:76–92. 10.1159/000060812 12061281

[42] Guénel P Laforest L Cyr D Févotte J Sabroe S Dufour C . Occupational risk factors, ultraviolet radiation, and ocular melanoma: a case-control study in France. Cancer Causes Control. (2001) 12:451–9. 10.1023/A:1011271420974 11545460

[43] Tenkate TD. Ocular ultraviolet radiation exposure of welders. Scand J Work Environ Health. (2017) 43:287–8. 10.5271/sjweh.3630 28295119

[44] McCarty CA Lee SE Livingston PM Taylor HR. Assessment of lifetime ocular exposure to UV-B: the Melbourne Visual Impairment Project. Modern Prob Ophthalmol. (1997) 27:9–13. 10.1159/000425642 8969956

[45] West SK Duncan DD Muñoz B Rubin GS Fried LP Bandeen-Roche K . Sunlight exposure and risk of lens opacities in a population-based study. The salisbury eye evaluation project. JAMA. (1998) 280:714–8. 10.1001/jama.280.8.714 9728643

[46] Moehrle M Garbe C. Personal UV-dosimetry by bacillus subtilis spore films. Dermatology. (2000) 200:1–5. 10.1159/000018306 10681605

[47] Moehrle M Dennenmoser B Garbe C. Continuous long-term monitoring of UV radiation in Professional mountain guides reveals extremely high exposure. Int J Cancer. (2003) 103:775–8. 10.1002/ijc.10884 12516097

[48] Carinou E Ferrari P Koukorava C Krim S Struelens L. Monte Carlo calculations on extremity and eye lens dosimetry for medical staff at interventional radiology procedures. Radiat Protect Dosimet. (2011) 144:492–6. 10.1093/rpd/ncq573 21212075

[49] Caracappa P Rhodes A Fiedler D. Multi-resolution voxel phantom modeling: a high-resolution eye model for computational dosimetry. Phys Med Biol. (2014) 59:5261. 10.1088/0031-9155/59/18/5261 25144465

[50] Santos MF Cassola V Kramer R Costa JV Andrade MEA Asfora VK . Development of a realistic 3D printed eye lens dosemeter using CAD integrated with Monte Carlo simulation. Biomed Phys Eng Exp. (2019) 6:015009. 10.1088/2057-1976/ab57bf 33438597

[51] Streicher J Culverhouse W Dulberg M Fornaro RJ. Modeling the anatomical distribution of sunlight. Photochem Photobiol. (2004) 79:40–7. 10.1111/j.1751-1097.2004.tb09855.x 14974714

[52] Vernez D Milon A Francioli L Bulliard JL Vuilleumier L Moccozet L. A numeric model to simulate solar individual ultraviolet exposure. Photochem Photobiol. (2011) 87:721–8. 10.1111/j.1751-1097.2011.00895.x 21223287

[53] Backes C Religi A Moccozet L Behar-Cohen F Vuilleumier L Bulliard JL . Sun exposure to the eyes: predicted UV protection effectiveness of various sunglasses. J Expos Sci Environ Epidemiol. (2019) 29:1. 10.1038/s41370-018-0087-0 30382242 PMC6803516

