# Erratum: Assessing human eye exposure to UV light: A narrative review

**DOI:** 10.3389/fpubh.2023.1183201

**Published:** 2023-03-21

**Authors:** Frontiers Production Office

**Affiliations:** Frontiers Media SA, Lausanne, Switzerland

**Keywords:** ocular exposure, ultraviolet radiation, UV, eye, ocular dosimetry

An omission to the funding section of the original article was made in error. The following sentence has been added: ”Open access funding was provided by the University of Geneva.”

The original article has been updated.

